# Acute cholecystitis with sepsis due to *Edwardsiella tarda*: a case report

**DOI:** 10.1186/s40792-023-01763-z

**Published:** 2023-10-24

**Authors:** Chisato Hara, Tetsuya Tanaka, Satoshi Nishiwada, Yuki Kirihataya, Atsushi Yoshimura

**Affiliations:** Department of Surgery, Minami-Nara General Medical Center, 8-1 Oaza-Fukugami Oyodo-cho, Yoshino-gun, Nara 638-8551 Japan

**Keywords:** *Edwardsiella tarda*, Acute cholecystitis, Sepsis, Antibiotics, Laparoscopy

## Abstract

**Background:**

*Edwardsiella tarda* (*E. tarda*) is a Gram-negative facultative anaerobe belonging to Enterobacteriales and is commonly isolated from fishes and reptiles. Infection due to *E. tarda* is uncommon among humans, with a reported human retention rate of 0.001%. It can cause sepsis in the elderly or those with pre-existing conditions such as liver failure, autoimmune disease, or malignancy. *E. tarda* is susceptible to many antibiotics; however, a high mortality rate (approximately 40%) has been reported with sepsis.

**Case presentation:**

A 65-year-old woman presented to our hospital with a chief complaint of fever and abdominal pain for 2 days. Her blood tests showed elevated inflammatory markers, and contrast-enhanced computed tomography showed distention and wall thickening of the gallbladder and inflammation of peri-gallbladder fat. Subsequently, a diagnosis of cholecystitis with systemic inflammatory response syndrome was made. Laparoscopic cholecystectomy was performed after starting antimicrobial therapy. Blood culture of samples obtained on admission were positive for *E. tarda*, which was also detected in bile juice culture. Therefore, she was diagnosed with bacteremia caused by *E. tarda*, and postoperative antimicrobial therapy was continued. The patient improved, and there were no complications.

**Conclusions:**

We experienced an extremely rare case of acute cholecystitis caused by *E. tarda*. Only a few cases of acute cholecystitis due to *E. tarda* have been reported. Furthermore, similar to this case, no previous study has reported the detection of *E. tarda* in both blood and bile cultures in acute cholecystitis cases. In addition to appropriate surgical intervention, continuous administration of antibiotics based on culture results resulted in a favorable outcome.

## Background

*Edwardsiella tarda* (*E. tarda*) is a Gram-negative rod-shaped bacterium that belongs to the Enterobacteriaceae family. *E. tarda* is isolated from fish and reptiles as a normal inhabitant. Although rare, it can be transmitted to humans through ornamental fish, pet turtles, snakes, catfish, and other animals, with a reported human carriage rate of 0.001% [1]. *E. tarda* may cause both intestinal and extraintestinal infections. Generally, intestinal infections are spontaneously resolved, whereas extraintestinal infections can lead to meningitis, liver abscess, necrotizing, fasciitis, and wound infection. Although sepsis caused by *E. tarda* is rare, it has a rapid course and a high fatality rate (approximately 40%) [2, 3]. Herein, we report a case of acute cholecystitis with sepsis caused by *E. tarda* wherein laparoscopic cholecystectomy was performed.

## Case presentation

**Fig. 1:**
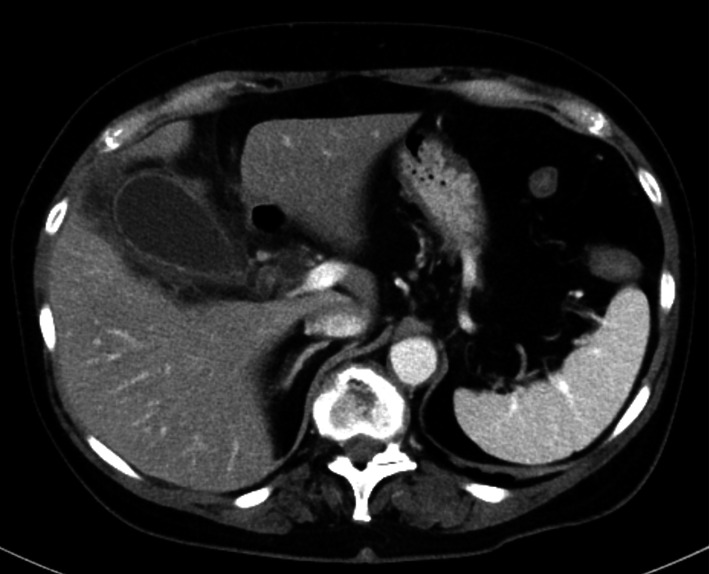
Contrast-enhanced computed tomography. Image shows gallbladder distention, wall thickening, and pericholecystic inflammation

**Fig. 2:**
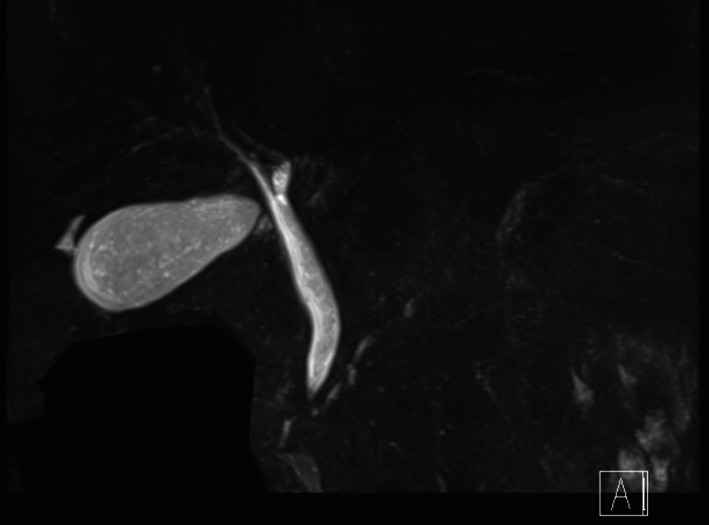
Magnetic resonance cholangiopancreatography. No gallbladder or common bile duct stones were found

**Fig. 3:**
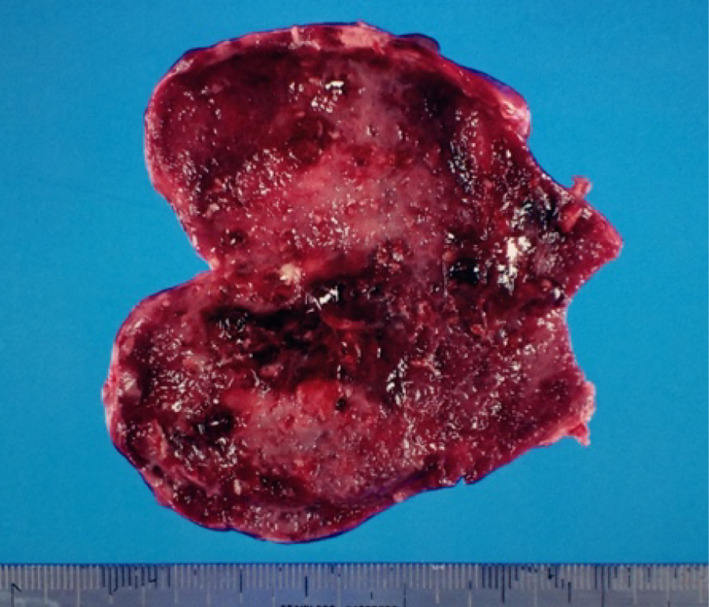
Specimen of surgically removed gallbladder. The gallbladder wall was partially necrotic. No stones were found in the gallbladder

**Fig. 4:**
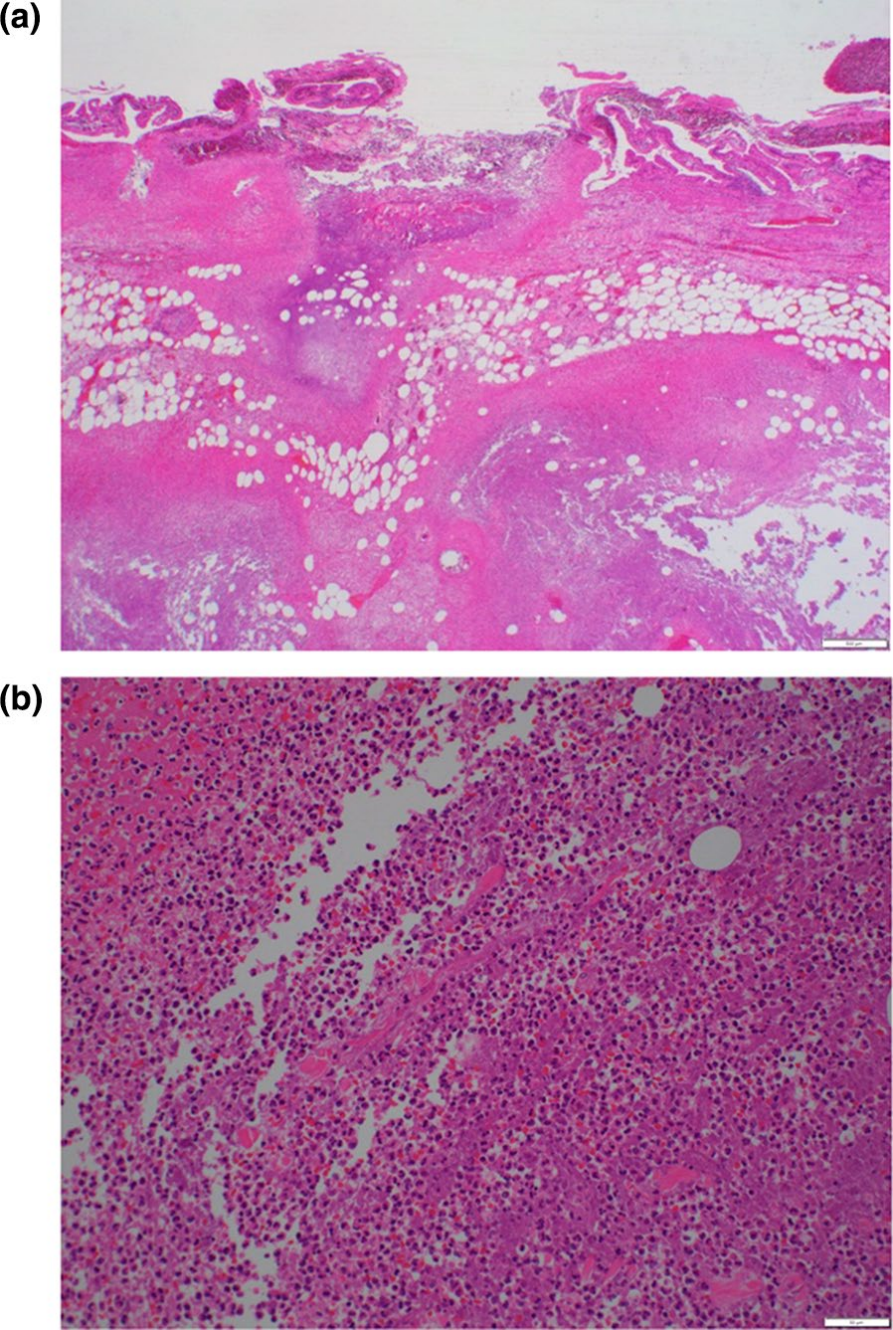
Photomicrograph of hematoxylin and eosin-stained gallbladder specimen. **a** Image shows full-thickness inflammation with partial degeneration, shedding of the gallbladder epithelium, and necrosis in some layers (magnification, 20 ×). **b** Image shows intense neutrophilic infiltration of the gallbladder epithelium (magnification, 200 ×)

**Fig. 5:**
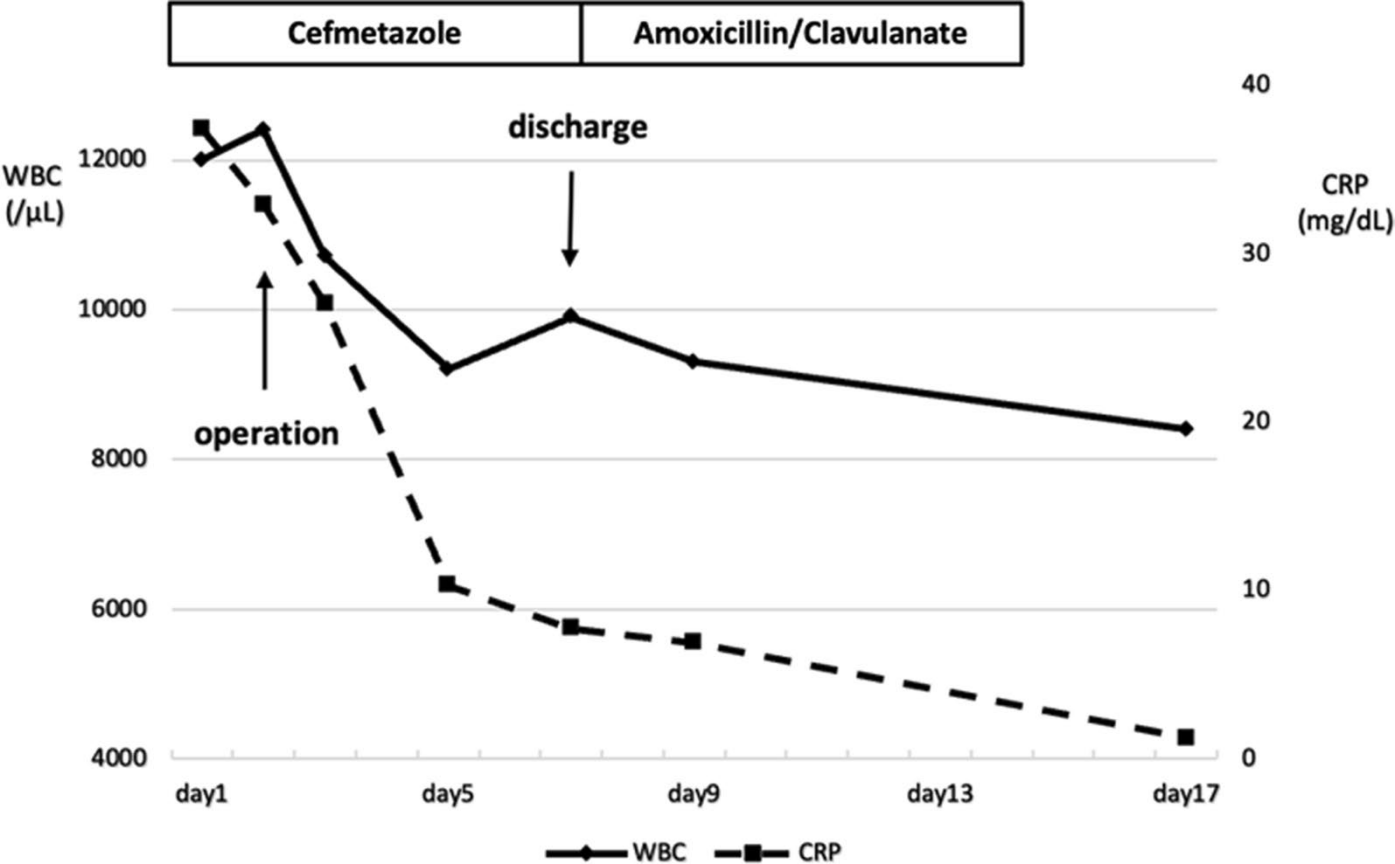
Changes in the number of white blood cells and C-reactive protein levels. Both white blood cell counts and C-reactive protein levels improved following surgery and antibiotic therapy. Cefmetazole was continued until the 7th postoperative day, and amoxicillin/clavulanate was continued until the 15th postoperative day. The patient was discharged on the 7th postoperative day and did not develop any complications

A 65-year-old woman presented to our hospital with fever and abdominal pain for 2 days. She had a history of asthma, depression, and irritable bowel syndrome. She had no history of keeping fish or other pets and had never traveled abroad. Upon presentation, her vital signs were as follows: temperature, 37.8 ℃, blood pressure, 103/84 mmHg, pulse rate, 124 beats/min, respiratory rate, 24 breaths/min, and oxygen saturation, 94% on room air. Her abdomen was flat and soft, but tenderness was noted in the right upper abdomen. Blood examination showed the following: leukocytes, 12,000/µL; and C-reactive protein, 37.40 mg/dL (Table 1). Contrast-enhanced computed tomography showed gallbladder distention, wall thickening, and inflammation of peri-gallbladder fat (Fig. 1). Magnetic resonance cholangiopancreatography did not show gallbladder or common bile duct stones (Fig. 2). Based on these tests, a diagnosis of acute cholecystitis with systemic inflammatory response syndrome was made. Our case was classified as moderate acute cholecystitis with marked local inflammatory findings (grade II) per the severity criteria of the Tokyo Guidelines 2018 Acute Cholecystitis [4]. After obtaining blood samples for culture, antibiotic therapy was started, and laparoscopic cholecystectomy was performed. Laparoscopy revealed an enlarged gallbladder; hence, puncture aspiration of bile, and decompression of the gallbladder were performed followed by laparoscopic cholecystectomy. Dark bloody bile was collected and submitted for culture examination. The wall of the removed gallbladder was partially necrotic, and no stones were found in the gallbladder (Fig. 3). Histopathological findings showed intense neutrophilic infiltration of all layers of the gallbladder epithelium and necrosis in some layers. At this time, the diagnosis was pyogenic and gangrenous cholecystitis (Fig. 4). Postoperatively, she had no complications. Results of blood and bile juice cultures were positive for *E. tarda* (Table 2). Based on the results of the antibiotic sensitivity test, cefmetazole was continued until the 7th postoperative day, and she was discharged home thereafter. After discharge, she continued receiving amoxicillin/clavulanate until postoperative day 15 without any complications (Fig. 5).

**Table 1:** The laboratory data

Hematology		Blood chemistry	
WBC	12,000/μL	TP	6.15 g/dL
Neutro	90.5%	Albumin	3.78 g/dL
Lymph	7.7%	BUN	17.8 mg/dL
Mono	1.5%	Creatinine	0.80 mg/dL
Eosino	0.2%	Sodium	137 mEq/L
Baso	0.1%	Potassium	3.7 mEq/L
RBC	452 × 10^4^/μL	Chloride	99 mEq/L
Hemoglobin	13.4 g/dL	Calcium	9.1 mg/dL
Hematocrit	41.5%	Uric acid	4.0 mg/dL
Platelet	17.3 × 10^4^/μL	AST	28 U/L
PT	84.5%	ALT	31 U/L
		ALP	129 U/L
		γ-GTP	129 U/L
		LDH	235 U/L
		T.Bil	2.07 mg/dL
		D.Bil	1.11 mg/dL
		CRP	37.40 mg/dL

**Table 2:** Antibiotic susceptibility of *E. tarda* from blood culture and bile culture

Antibiotic	Blood culture		Bile culture	
	MIC(μg/mL)		MIC(μg/mL)	
GM	≤ 1	S	≤ 1	S
ST	≤ 20	S	≤ 20	S
AMK	≤ 2	S	≤ 2	S
CAZ	≤ 1	S	≤ 1	S
CMZ	≤ 1	S	2	S
CTM	≤ 8	S	≤ 8	S
CTX	≤ 1	S	≤ 1	S
FOM	≤ 16	S	≤ 16	S
IPM	≤ 0.25	S	≤ 0.25	S
ABPC	≤ 2	S	≤ 2	S
CFPM	≤ 1	S	≤ 1	S
CPDX	≤ 0.25	S	0.5	S
CPFX	≤ 0.25	S	≤ 0.25	S
AMPC/CVA	≤ 2	S	≤ 2	S
LVFX	≤ 0.12	S	≤ 0.12	S
MEPM	≤ 0.25	S	≤ 0.25	S
MINO	≤ 1	S	≤ 1	S
PIPC	≤ 4	S	≤ 4	S

Determined based on the 29th edition of Clinical and Laboratory Standards Institute (CLSI) document M100

*GM* gentamicin, *ST* sulfamethoxazole–trimethoprim, *AMK* amikacin, *CAZ* ceftazidime, *CMZ* cefmetazole, *CTM* cefotiam, *CTX* cefotaxime, *FOM* fosfomycin, *IPM* imipenem, *ABPC* ampicillin, *CFPM* cefepime, *CPDX* cefpodoxime, *CPFX* ciprofloxacin, *AMPC/CVA* amoxicillin/clavulanate, *LVFX* levofloxacin, *MEPM* meropenem, *MINO* minocycline, *PIPC* piperacillin, *MIC* minimum inhibitory concentration, *S* sensitive

## Discussion

*E. tarda* is a Gram-negative facultative anaerobe belonging to Enterobacteriales and is isolated from turtles, fish, snakes, and lizards associated with fresh and saltwater [5–7]. *E. tarda* is a known pathogen of fish and reptiles and is uncommonly reported in humans. However, *E. tarda* can be transmitted to humans through contact with reptiles, amphibians, and other carriers of the disease as well as through ingestion of raw fish [8]. *E. tarda* infection in humans is rare, with a human retention rate of 0.001% [1]. A search of PubMed using “*Edwardsiella tarda*” and “acute cholecystitis” as search terms yielded several cases. Among them, only 12 cases of acute cholecystitis and *Edwardsiella tarda*-induced bacteremia have been reported [9–12]. Our case and its 12 cases are summarized in Table 3. The study population was relatively older, with a mean age of 74.6 years, and comprised 7 males and 6 females. In addition to antimicrobial therapy, we performed invasive gallbladder procedures such as PTGBD in 3 patients and surgery in 5 patients. Furthermore, there are no reports of *E. tarda* being detected in both blood and bile cultures in cases of acute cholecystitis, similar to the present case.

**Table 3:** Detailed characteristics of patients with acute cholecystitis and *Edwardsiella tarda*-induced bacteremia

Reference	Age/sex	Interval from the onset	Organisms from blood culture	Organisms from bile culture	Underlying disease	Comorbidities	Coexisting gallstones	Treatment	Treatment duration
Kamiyama et al. [9]	70/F	Unknown	*E. tarda*	None	None	Cholangitis, sepsis	Unknown	Unknown	8 days
	75/F	Unknown	*E. tarda*	*Klebsiella *sp.*, E. coli*	None	None	Unknown	Unknown	3 days
	65/M	Unknown	*E. tarda, Streptococcus gallolyticus*	None	Gastric cancer, gallstone	Cholangitis	Yes	Unknown	13 days
Tonosaki K et al. [10]	93/F	Within 24 h	*E. tarda*	Unknown	Resection of left breast cancer, total hysterectomy for uterine fibroids, shingles	Sepsis, disseminated intravascular coagulation	Yes	Antimicrobials	35 days
Hasegawa K et al. [11]	82/M	Unknown	*E. tarda*	Unknown	Gallstone	None	Yes	PTGBD, antimicrobials	Unknown
	75/M	Unknown	*E. tarda*	Unknown	Colorectal cancer (pre-existing condition), gallstone	None	Unknown	Surgery, antimicrobials	14 days
	77/M	Unknown	*E. tarda*	Unknown	Gallstone, chronic liver disease (HCV)	None	Yes	ERCP, antimicrobials	9 days
	68/M	Unknown	*E. tarda*	Unknown	Gallstone, Chronic liver disease (HBV)	None	Yes	Surgery, antimicrobials	9 days
	74/M	Unknown	*E. tarda*	Unknown	Prostate cancer(surgery), gallstone, cholangitis, cardiovascular disease, hypertension, chronic lung disease	Cholangitis, sepsis	Yes	Surgery, antimicrobials	15 days
	85/F	Unknown	*E. tarda*	Unknown	Gallstone, cholangitis, hypertension, stroke	None	Yes	PTGBD, antimicrobials	10 days
	77/F	Unknown	*E. tarda*	Unknown	Autoimmune hepatitis, lung cancer (chemotherapy), cholecystitis	None	Unknown	PTGBD, antimicrobials	11 days
Yue Ding et al. [12]	64/M	3 days	*E. tarda*	Unknown	Gallstones, bile duct stones, Chronic liver disease (HBV)	Cholangitis, sepsis	Yes	Surgery, antimicrobials	7 days
Our case	65/F	2 days	*E. tarda*	*E. tarda, Klebsiella pneumoniae*	Asthma, depression, irritable bowel syndrome	Sepsis	No	Surgery, antimicrobials	7 days

*ERCP* endoscopic retrograde cholangiopancreatography, *PTGBD* percutaneous transhepatic gallbladder drainage, *HBV* hepatitis B virus, *HCV* hepatitis C virus

Intestinal infections account for approximately 80% of all *E. tarda* infections [13]. *E. tarda* is biochemically similar to Salmonella; it causes low-grade fever and intermittent watery diarrhea, similar to Salmonella enteritis [8, 9]. Generally, enteritis caused by *E. tarda* often resolves spontaneously with symptomatic treatment and is rarely clinically severe. However, intra-abdominal abscess, cholecystitis, cholangitis, cellulitis, necrotizing fasciitis, meningitis, and osteomyelitis have been reported as extraintestinal infections, although the number of reports is small [9–11, 14–18]. Meanwhile, bacteremia is rare, occurring in less than 5% of all cases of *E. tarda* infections [8]. However, the mortality from bacteremia caused by *E. tarda* is 44.6% [3]. Risk factors for severe *E. tarda* infection include age (≥ 65 years) and a history of underlying diseases such as malignancy, autoimmune disease, liver disease, and diabetes mellitus [3, 9, 11]. In these patients, mortality rates were reportedly more than four times higher especially in patients with cirrhosis [3, 19]. Additionally, deaths have been reported in cases of sepsis associated with soft tissue infections such as necrotizing fasciitis, which may be due to the difficulty in completely removing the infected lesion [3].

Regarding treatment, extraintestinal infections generally require antibiotic therapy. *E. tarda* is sensitive to several antibiotic agents, including β-lactams, aminoglycosides, quinolones, and tetracyclines [20]. However, in cases of bacteremia, which can be severe, it is necessary to continue antibiotic therapy even after symptoms improve. Therefore, it is important to administer antibiotics after culture testing to determine the appropriate antibiotic therapy. In our case, blood cultures were obtained before antibiotics were administered, and intraoperative bile cultures were also obtained. Results confirmed *E. tarda* infection, allowing treatment with the appropriate antibiotic agents. Additionally, due to the importance of complete removal of the infected lesion, surgical intervention would be appropriate.

Regarding the route of infection in this case, the patient had no history of keeping fish or reptiles as pets, and the possibility of contact infection was considered low. However, because of her habit of eating raw seafood on a daily basis, the possibility of infection by oral ingestion was considered possible. After the diagnosis of *E. tarda* infection, fecal culture was performed to reveal intestinal commensal bacterial; however, *E. tarda* was not detected because culture was performed after antibiotic therapy was started. Although the reported human retention rate is 0.001% [1], the possible presence of E. tarda cannot be ruled out due to the patient’s history of recurrent diarrhea, which was caused by irritable bowel syndrome. In our case, there were no gallbladder stones that could have caused cholecystitis. Gallbladder stones are the most common cause of acute cholecystitis development. Cholecystitis develops due to gallbladder duct obstruction and bile congestion caused by the fitting of a stone, which damages the mucosa of the gallbladder. Conversely, acute acalculous cholecystitis can occur in 3.7–14% patients with acute cholecystitis [21, 22]. Risk factors of cholecystitis include surgery, trauma, infection, burns, and transvenous nutrition [23, 24]. However, these risk factors were not relevant in our case. As diarrhea was previously observed in this case, the patient developed a retrograde biliary infection due to enteritis-induced increased intestinal pressure, which occurred due to irritable bowel syndrome; this could have further led to cholecystitis and then to bacteremia. There have been many reports of patients with a poor prognosis caused by *E. tarda* sepsis; however, in our case, she had a favorable prognosis. This may be attributed to the patient’s age, early and appropriate surgical intervention to completely remove the infected lesion, and continued systematic administration of antimicrobials based on the culture results, all of which prevented recurrence.

## Conclusion

We report a case of sepsis secondary to acute cholecystitis caused by *E. tarda* that was treated with laparoscopic cholecystectomy. Although *E. tarda* rarely infects humans, it can occasionally cause sepsis, which can be severe, and result in death. Therefore, this infection needs to be recognized, and appropriate therapeutic interventions should be implemented early.

## Data Availability

The dataset supporting the conclusions of this article is available in the manuscript.

## Abbreviations

*E. tarda*: *Edwardsiella tarda*
WHO: World Health Organization
WBC: White blood cell count
RBC: Red blood cell count
PT: Prothrombin time
TP: Total protein
BUN: Blood urea nitrogen
AST: Aspartate aminotransferase
ALT: Alanine aminotransferase
ALP: Alkaline phosphatase
γ-GTP: γ-Glutamyltranspeptidase
LDH: Lactate dehydrogenase
T.Bil: Total bilirubin
D.Bil: Direct bilirubin
CRP: C-reactive protein
MIC: Minimum inhibitory concentration
CLSI: Clinical and Laboratory Standards Institute
GM: Gentamicin
ST: Sulfamethoxazole–trimethoprim
AMK: Amikacin
CAZ: Ceftazidime
CMZ: Cefmetazole
CTM: Cefotiam
CTX: Cefotaxime
FOM: Fosfomycin
IPM: Imipenem
ABPC: Ampicillin
CFPM: Cefepime
CPDX: Cefpodoxime
CPFX: Ciprofloxacin
AMPC/CVA: Amoxicillin/clavulanate
LVFX: Levofloxacin
MEPM: Meropenem
MINO: Minocycline
PIPC: Piperacillin
S: Sensitive
ERCP: Endoscopic retrograde cholangiopancreatography
PTGBD: Percutaneous transhepatic gallbladder drainage
HBV: Hepatitis B virus
HCV: Hepatitis C virus

## References

[1] Onogawa T, Terayama T, Zen-yoji H, Amano Y, Suzuki K (1976). Distribution of *Edwardsiella tarda* and hydrogen sulfide-producing *Escherichia coli* in healthy persons. Kansenshogaku Zasshi.

[2] Wang IK, Kuo HL, Chen YM, Lin CL, Chang HY, Chuang FR (2005). Extraintestinal manifestations of *Edwardsiella tarda* infection. Int J Clin Pract.

[3] Hirai Y, Asahata-Tago S, Ainoda Y, Fujita T, Kikuchi K (2015). *Edwardsiella tarda* bacteremia: a rare but fatal water- and foodborne infection: review of the literature and clinical cases from a single centre. Can J Infect Dis Med Microbiol.

[4] Yokoe M, Hata J, Takada T, Strasberg SM, Asbun HJ, Wakabayashi G (2018). Tokyo Guidelines 2018: diagnostic criteria and severity grading of acute cholecystitis (with videos). J Hepatobiliary Pancreat Sci.

[5] Nagel P, Serritella A, Layden TJ (1982). *Edwardsiella tarda* gastroenteritis associated with a pet turtle. Gastroenterology.

[6] Vandepitte J, Lemmens P, De Swert L (1983). Human Edwardsiellosis traced to ornamental fish. J Clin Microbiol.

[7] Sakazaki R (1965). A proposed group of the family *Enterobacteriaceae*, the Asakusa group. Int Bull Bacteriol Nomencl Taxon.

[8] Janda JM, Abbott SL (1993). Infections associated with the genus *Edwardsiella*: the role of *Edwardsiella tarda* in human diseases. Clin Infect Dis.

[9] Kamiyama S, Kuriyama A, Hashimoto T (2019). *Edwardsiella tarda* bacteremia, Okayama, Japan, 2005–2016. Emerg Infect Dis.

[10] Tonosaki K, Yonenaga K, Mikami T, Mizuno T, Oyama S (2021). Acute cholecystitis, sepsis, and disseminated intravascular coagulation caused by *Edwardsiella tarda* in an elderly woman. Tokai J Exp Clin Med.

[11] Hasegawa K, Kenya M, Suzuki K, Ogawa Y (2022). Characteristics and prognosis of patients with *Edwardsiella tarda* bacteremia at a single institution, Japan, 2005–2022. Ann Clin Microbiol Antimicrob.

[12] Ding Y, Men W (2022). A case report and review of acute cholangitis with septic shock induced by *Edwardsiella tarda*. Ann Clin Microbiol Antimicrob.

[13] Jordan GW, Hadley WK (1969). Human infection with *Edwardsiella tarda*. Ann Intern Med.

[14] Pham K, Wu Y, Turett G, Prasad N, Yung L, Rodriguez GD (2021). *Edwardsiella tarda*, a rare human pathogen isolated from a perihepatic abscess: implications of transient versus long term colonization of the gastrointestinal tract. IDCases.

[15] Yamamuro T, Fukuhara A, Kang J, Takamatsu J (2019). A case of necrotizing fasciitis following *Edwardsiella tarda* septicemia with gastroenteritis. J Infect Chemother.

[16] Yamanoi K, Yasumoto K, Ogura J, Hirayama T, Suginami K (2018). A case of pelvic abscess caused by *Edwardsiella tarda* followed by laparoscopic resection of a hematoma derived from cesarean section. Infect Dis.

[17] Miyazawa Y, Murakami K, Kizaki Y, Itaya Y, Takai Y, Seki H (2018). Maternal peripartum septic shock caused by intrauterine infection with *Edwardsiella tarda*: a case report and review of the literature. J Obstet Gynaecol Res.

[18] Makino T, Sugano I, Kamitsukasa I (2018). An autopsy case of *Edwardsiella tarda* meningoencephalitis. Case Rep Neurol.

[19] Arvaniti V, D’Amico G, Fede G, Manousou P, Tsochatzis E, Pleguezuelo M (2010). Infections in patients with cirrhosis increase mortality four-fold and should be used in determining prognosis. Gastroenterology.

[20] Stock I, Wiedemann B (2001). Natural antibiotic susceptibilities of *Edwardsiella tarda*, *E. ictaluri*, and *E. hoshinae*. Antimicrob Agents Chemother.

[21] Wang AJ, Wang TE, Lin CC, Lin SC, Shih SC (2003). Clinical predictors of severe gallbladder complications in acute acalculous cholecystitis. World J Gastroenterol.

[22] Ryu JK, Ryu KH, Kim KH (2003). Clinical features of acute acalculous cholecystitis. J Clin Gastroenterol.

[23] Laurila J, Syrjälä H, Laurila PA, Saarnio J, Ala-Kokko TI (2004). Acute acalculous cholecystitis in critically ill patients. Acta Anaesthesiol Scand.

[24] Theodorou P, Maurer CA, Spanholtz TA, Phan TQ, Amini P, Perbix W (2009). Acalculous cholecystitis in severely burned patients: incidence and predisposing factors. Burns.

